# Pharmacoeconomic evaluation of an antimicrobial stewardship program in a tertiary care hospital of Sindh, Pakistan

**DOI:** 10.12669/pjms.42.6.15299

**Published:** 2026-06

**Authors:** Arslan Ahmer, Muhammad Ali Ghoto, Mah’d Musa Eid Abu Dhair, Lareb Asad

**Affiliations:** 1 Arslan Ahmer, Faculty of Pharmaceutical Sciences, People’s University of Medical and Health Sciences for Women,Nawabshah, Sindh, Pakistan; 2 Muhammad Ali Ghoto, Faculty of Pharmacy, University of Sindh, Jamshoro, Sindh, Pakistan; 3 Mah’d Musa Eid Abu Dhair, Department of Pharmacy Practice, Faculty of Pharmacy, University of Sindh, Jamshoro, Sindh, Pakistan; 4 Lareb Asad, Department of Pathology, People’s University of Medical and Health Sciences for Women,Nawabshah, Sindh, Pakistan

**Keywords:** Antimicrobial Stewardship Program, Antimicrobial Resistance, ASP Implementation, ASP Pakistan, Pharmacoeconomic Evaluation

## Abstract

**Background & Objective:**

Escalation of antimicrobial resistance (AMR) globally has led to increased use of healthcare resources, increased lengths of hospital stay, and increased costs of treatment. Our objective was to determine the economic impact of Antimicrobial Stewardship Program (ASP) implementation on antimicrobial therapy cost and its correlation with demographic factors.

**Methodology:**

A prospective, quasi - experimental, pharmacoeconomic study was carried out for an eighteen months period (July 2023 to December 2024) in People’s Medical College Hospital Nawabshah. Three thousand patients were recruited based on Cochran’s formula (1500 in each study phase i.e. pre and post ASP implementation). Analyses were done from hospital perspective, focusing solely on direct costs of antimicrobial treatment. Demographic subgroups were analyzed for their effect on the costs. Statistical procedure included descriptive statistics, independent t tests, chi square tests and multivariate linear regression. A threshold for significance, p<0.05 was considered.

**Results:**

Mean antimicrobial-therapy costs dropped significantly after ASP implementation in both medical and surgical wards, by a total of 17%. In 18-29 aged patients, reduction in cost was seen 16.2% followed by 18% in 30-49 years, 17.1% in 50-69 years and 14.7% in ≥70 years patients. Multivariate regression analysis showed that age, type of ward and gender were independent determinants of cost and the implementation of ASP was the strongest cost reducing predictor identified.

**Conclusion:**

ASP has potential of providing much greater pharmacoeconomic benefits in hospitals. There is significant influence of demographic factors in particular, age and ward type have critical role on costs of antimicrobials.

## INTRODUCTION

Antimicrobial resistance represents one of the greatest contemporary threats to global public health. In low and middle income countries (LMICs), the AMR burden is seriously disproportionate due to the availability of antibiotics without a prescription, lack of regulatory control, inadequate infection control measures and irrational prescriptions practices.^1^

Pakistan, having one of the highest antibiotic consumption rates in the world, is especially at risk of the economic and clinical consequences of AMR.^2^ Surveillance data from both the Global Antimicrobial Resistance Surveillance System (GLASS) show extremely high levels of resistance to common pathogens such as *Escherichia coli*, *Klebsiella pneumoniae* and *Acinetobacter baumannii*.^3^ These patterns of resistance are reflected in prolonged hospital admissions, higher mortality and high healthcare costs - especially in the tertiary care setting.

Antimicrobial Stewardship Programs (ASPs) have appeared as one of the pillars of intervention in combating AMR. Recommendations from the World Health Organization (WHO), the Centers for Disease Control and Prevention (CDC) and the Infectious Diseases Society of America (IDSA) have reached a consensus that ASPs are essential measures to optimize the use of antibiotics, reduce resistance and improve patient outcomes. Beyond clinical improvements, ASPs have shown an increasing number of positive economic impacts such as preventing unnecessary antimicrobial utilization, reducing the length of stay, and lowering the cost of therapy.^4^-^7^

Despite increasing evidence worldwide, the pharmacoeconomic impact of ASPs in Pakistan particularly in settings of public tertiary institutions is rarely reported. Existing studies mostly examine clinical or microbiological outcomes with little consideration of direct cost savings or the demographic determinants of expenditure.^8^ Moreover, the limited number of cost-evaluations that exist are generally focused on urban areas, private hospitals or resource-rich hospitals, thus there is a critical gap in the evidence in rural and resource-constrained public environments.

This study aimed to a detailed pharmacoeconomic evaluation by assessing the impact of antimicrobial therapy costs before and after the implementation of ASP in a tertiary care hospital in Nawabshah, Sindh, and examines the relationship between demographic factors and cost outcomes.

## METHODOLOGY

A hospital based, prospective, quasi-experimental study was conducted over eighteen months to study from July 2023 to December 2024 in People’s Medical College Hospital (PMCH), Nawabshah, which is a public sector tertiary care teaching hospital of over 500 beds situated in rural Sindh. The hospital has a patient population of over 1.8 million and has departments of medicine, surgery, paediatric, obstetrics/gynecology, and intensive care units. The site was selected for its high volume of antimicrobial consumption, availability of baseline data for pharmacy and microbiology, and institutional interest in support of implementation of ASPs.

### Ethical considerations:

Ethical approval was taken by Institutional Bioethics Committee, University of Sindh, Jamshoro (Approval No. ORIC/SU/1517, Dated: June 19, 2023) and hospital consent was obtained from the Medical Superintendent of People’s Medical College Hospital (Ref No: PMCHN (SBA)/16012, Date: June 23, 2023). Informed consent was secured, and confidentiality was maintained.

### Study Population and Sample size:

The study population included adult inpatients (≥18 years), who received at least one systemic antimicrobial agent during their admission to either medical or surgical wards. Exclusion criteria included infants and children, patients discharged within 24 hours, patients with incomplete medical records and patients enrolled in concurrent clinical trials.

- Three thousand (3000) patients were used, calculated through Cochran’s formula.^9^
- Pre-ASP phase (Baseline): 1,500 patients (July–December 2023)
- Post-ASP phase (Intervention): 1,500 patients (April–December 2024)
- Whereas, Jan–Mar 2024 was ASP introduction/implementation phase, so patients’ data were not observed during this phase.

### Patient flow and missing data:

A total of 3,248 patients were screened, of whom 3,000 met inclusion criteria and were included in the final analysis (1,500 pre-implementation; 1,500 post-implementation). Exclusions were primarily due to incomplete records or protocol deviations. Missing data were minimal and managed using complete case analysis with sensitivity testing to ensure robustness of cost estimates.

### ASP Intervention:

- The ASP was planned and implemented by a multidisciplinary team of physicians, pharmacists, microbiologists and infection control personnel. Key interventions included:
- Prospective audit and feedback within 72 hours of antimicrobial initiation
- Formulary restriction of broad-spectrum antibiotics
- Development of local treatment guidelines
- Promotion of diagnostic stewardship (culture testing before initiation)
- WHO defined daily dose (DDD) metrics
- Educational sessions for prescribers and nursing staff

### Pharmacoeconomic Evaluation/Analysis:

A **cost-minimization analysis** (CMA),^10^ was applied and supported by a **cost-consequence framework.** Economic evaluation took the perspective of the hospital, and focused only on the direct costs of antimicrobial therapy. The cost of each patient was calculated based on pharmacy dispensing records, cross-checked with patient charts. Expenses were expressed in terms of Pakistani Rupees (PKR).

Cost-minimization analysis was deemed appropriate as both treatment strategies demonstrated equivalent clinical effectiveness. Equivalence was established based on comparable clinical cure rates, microbiological outcomes, mortality, readmission rates, etc. Statistical testing confirmed absence of significant differences across all endpoints, thereby satisfying the fundamental assumption required for CMA.

Direct medical costs were calculated by multiplying resource utilization by corresponding unit costs. Unit costs were obtained from hospital procurement records and finance department tariffs. All costs were standardized to 2023-2024 PKR to account for inflation. The total cost per patient represented the sum of antimicrobial acquisition costs, laboratory investigations, hospital stay, diagnostic procedures, and supportive medications. Mean per-patient costs were compared between study groups under the assumption of equivalent clinical outcomes, consistent with the principles of cost-minimization analysis.

One-way sensitivity analysis was conducted by varying key cost parameters (antibiotic acquisition cost and length of stay cost) by ±20% to evaluate robustness of economic outcomes.

**CMA Formula**^22^: Δ*C ═ C_A_ - C_B_*

Where, Δ*C* represents cost difference and *C_A_, C_B_* represent the total costs of intervention A and B, respectively.

### Data collection:

A standardized questionnaire was used to collect the data as data collection tool, which consist the following sections:

- Demographic data: age, gender, ward type
- Cost data: total cost of antimicrobial therapy per patient

### Statistical analysis:

Data were analyzed using IBM SPSS version 28.

- Descriptive statistics (mean, SD, frequencies) were used for demographic/baseline characteristics.
- Independent t-tests assessed differences in mean costs pre- and post-ASP.
- Chi-square tests analyzed the distribution of cost categories across groups.
- Linear regression evaluated predictors of antimicrobial therapy cost.
- Statistical significance was set at p < 0.05.

## RESULTS

The findings provide an extensive pharmacoeconomic evaluation of the implementation of ASPs with an emphasis on patient demographic characteristics and direct costs of antimicrobial therapy. Analyses were descriptive statistics, comparative cost analyses, categorical distribution, and inferential statistics to identify cost predictors.

The demographic distribution was statistically comparable for groups of pre- and post-implementation ASP. Average patient age was similar between phases indicating a similar disease burden and profile of the patient. In gender distribution, the male gender reported 54.2%. Medical wards dealt with a higher number of patients than surgical in both phases, reflecting the case mix of the hospital, to ensure that comparisons of costs were not confounded by changes in demographics (Table-I).

**Table-I:** Demographic Characteristics of Study Population (N = 3,000).

Variable	Pre-ASP (n=1500)	Post-ASP (n=1500)	Total (n=3000)
Mean Age (years)	48.3 ± 16.4	47.9 ± 15.8	48.1 ± 16.1
Gender (Male/Female)	820 / 680	805 / 695	1,625 / 1,375
Ward Type (Medical/Surgical)	880 / 620	870 / 630	1,750 / 1,250

A statistically significant cost reduction in antimicrobial therapy mean was evident after the ASP implementation. Medical wards had a greater reduction 18.4% compared with surgical wards 15.4%, possibly indicating superior efficacy of stewardship interventions in general medical settings. The overall 17% reduction in antimicrobial costs was a significant economic savings, especially in a public hospital setting with limited resources (Table-II).

**Table-II:** Mean Antimicrobial Cost per Patient (PKR).

Ward	Pre-ASP (Mean ± SD)	Post-ASP (Mean ± SD)	% Reduction	p-value
Medical Wards	6,350 ± 1,320	5,180 ± 1,060	-18.4%	<0.001
Surgical Wards	7,120 ± 1,480	6,020 ± 1,210	-15.4%	<0.001
Overall Mean	6,680 ± 1,420	5,600 ± 1,135	-17.0%	<0.001

All age groups had substantial reductions in costs after implementation ASP, although these savings were most substantial for the 30-49 year age group (18% reduction). This pattern is better targeting of empiric therapy and in this demographic antibiotic choice is more careful. Patients aged ≥70 years consistently had the highest mean costs, both pre- and post-intervention, possibly because of multiple comorbidities, polypharmacy and length of stay (Table-III).

**Table-III:** Mean Antimicrobial Cost by Age (PKR).

Age Group (years)	Pre-ASP	Post-ASP	% Change	p-value
18–29	5,860	4,910	-16.2%	0.003
30–49	6,430	5,270	-18.0%	<0.001
50–69	6,980	5,790	-17.1%	<0.001
≥70	7,220	6,160	-14.7%	0.007

Following ASP implementation, a significant redistribution of patients into lower cost categories was reported. The patients’ proportion having therapy cost below PKR 5,000 almost doubled, indicating an increase in affordability. On the other hand, the dataset decreased in the patients’ proportion with high-cost prescriptions (>7500 PKR) to over 11%. It is through these shifts that ASPs are playing an important role in encouraging cost-effective prescribing and reducing the financial burden on the public health system (Table-IV).

**Table-IV:** Cost Category Distribution (PKR) of Patients.

Category (PKR)	Pre-ASP % (n)	Post-ASP % (n)	χ^2^	p-value
<5,000	18.3% (275)	34.1% (512)	118.5	<0.001
5,001–7,500	52.6% (789)	48.3% (725)		
>7,500	29.1% (436)	17.6% (263)		

The antimicrobial stewardship initiative produced sustained cost containment across wards and months, achieving measurable institutional savings (Table-V).

**Table-V:** Economic Indicators in Months before and after ASP Implementation.

Indicator	Pre-ASP	Post-ASP	% Change	p-value
Total antibiotic expenditure (PKR, per six months)	21.7 million	17.9 million	–17.5 %	p < 0.001
Average monthly antibiotic expenditure (PKR)	3.42 million	2.98 million	–12.9 %	p < 0.005

Multivariate linear regression showed the phase of the ASP intervention to be the most significant independent predictor of reduced cost of antimicrobial therapy (β = -1,080.3; p < 0.001). Age added incremental cost increases, with every year incremental added costing about 22 PKR. Admission to a surgical ward was associated with an additional PKR 810 higher cost, which could represent prophylactic and empiric antibiotic overuse. Female gender showed a small reduction in cost, however, underlying clinical factors are likely to be contributory rather than prescribing bias. This model does strengthen the ability of ASPs to contain costs when adjusted for key patient variables (Table-VI).

**Table-VI:** Linear Regression – Predictors of Antimicrobial Cost (PKR).

Variable	β Coefficient	95% CI	p-value
Age (per year)	+22.4	18.3 to 26.5	<0.001
Surgical Ward	+810.5	710.2 to 910.8	<0.001
Female Gender	-140.7	-240.5 to -40.9	0.005
Post-ASP Phase	-1,080.3	-1,180.5 to -980.1	<0.001

## DISCUSSION

People’s Medical College Hospital (PMCH) in Nawabshah, Pakistan implemented an Antimicrobial Stewardship Program (ASP) that reduced average costs of patient therapy by 17%. The average cost per patient decreased from PKR 6,680 (± 1,420) to PKR 5,600 (± 1,135) (p < .001). Total savings to the institution for total antimicrobials were sustained at 17.5% over six months (from PKR 21.7 million to 17.9 million; p < .001). All pharmacoeconomic savings resulted from cost-minimization analyses that assumed equivalent clinical outcomes and demonstrated the ability of multidisciplinary ASP interventions, including prospective audit with feedback to prescribers, formulary restrictions, local guidelines, diagnostic stewardship, and prescriber education, to contribute to cost-saving in resource-limited environments. A post-ASP phase was identified as having the greatest negative association with cost (β = -1,080.3 PKR; 95% CI: -1,180.5 to -980.1; p < .001). Multivariate regression analyses further demonstrated that increasing age (PKR +22.4 for each year; p < .001) and surgical ward admission (+810.5 PKR; p < .001) were significant predictors of cost increases, while being female resulted in slightly lower costs (-140.7 PKR; p = 0.005).

The results of this study support and exceed recent economic evaluations of ASPs done within low- and middle-income nations, where cost reductions are usually estimated at around 10-15%, but are often complicated by poor implementation (in particular due to urban bias). For example, a 2023 analysis of the situation within tertiary care hospitals in Karachi found that the main method used to achieve the goals of the ASP was through many prospective audits and feedbacks (the same method used by the PMCH), and that of the two private hospitals operating with structured ASPs (H1 and H2), neither one achieved pharmacy-driven dose optimization and IV-to-PO conversion with documented cost metrics for either hospital (strong private sector leadership is a contributing factor).^11^ The PMCH demonstrates how successfully public sector programs can be scaled up to include rural health care environments through the pharmacist-led consult program provided by PMCH, using renal function, pharmacokinetics, and site specific susceptibility information, as compared to the limited experience of the other hospitals and outpatient clinics. A similar grounded theory study using pharmacist-led AMS in Pakistan in 2024 supported both dose adjustment and automatic stop orders for broad-spectrum antibiotics and documented broad-spectrum antibiotic use reductions of as much as 20%, but did not include the direct cost impact of these adjustments. In contrast, the PMCH experienced a reduction of 18.4% of its total medical ward costs as a result of these pharmacy-led consult programs, thereby establishing its effectiveness and economic viability as an effective strategy for managing these high burden health care needs.^12^

Demographic stratification showed detailed costs: greatest reduction happened in 30-49 age range (18%; p < 0.001), but lower reduction in 50-69 age range (17.1%) with further reduced rates in those >= 70 (14.7%; p = 0.007). Increased effects from polypharmacy and/or altered dosing due to numerous concomitant diagnoses in older patients when compared to younger patients may have contributed this age-specific difference. Similar age-specific trends were reported by physicians in Pakistan through a qualitative multicenter study in 2023 that found older patients were disproportionately affected by empiric broad spectrum prescribing of antimicrobials due to delayed receipt of cultured specimens and limited faith in laboratory results, and that this created increased costs from longer hospital stays (15-25%). PMCH’s diagnostic stewardship changed this significantly, and there was more than a twofold increase in the percentage of patients charged less than PKR 5,000 (18.3% to 34.1%; χ^2^ = 118.5; p < 0.001) and a larger percentage of patients being charged more than PKR7,500 as compared to pre-ASP time periods; costs per patient in PMCH were found to be associated with diagnosis coding according to ASP procedures, and demonstrate PMCH’s formulary restrictions as a successful intervention addendum to resolve the problems.^13^ Comparative costs between wards (i.e. 18.4% for medicine and 15.4% for surgery) and pre-ASP costs (i.e. 20-30%) reflect that the use of surgical prophylaxis has resulted in excess costs at district hospitals, thereby further validating PMCH formulary restrictions as a targeted intervention.^14^

PMCH developed a ward-level measures and a 12.9% reduction in monthly drug costs (from PKR 2.98 million) allowed PMCH to develop this information to support and assess performance in regard to the SHEA/IDSA core measures of performance for process and outcome monitoring.^10^ A global 2023 WHO point prevalence survey of LMICs (low-middle income countries) reports average of 14% cost savings using similar methods; however, PMCH achieved a cost savings of 17%, which exceeds U.S. federal IDSA cohort benchmarks (15% to 22%) for cost savings adjusted for inflation-adjusted PKR and using a ±20% sensitivity determination.^13^,^15^

In light of Pakistan’s AMR crisis (1.27 million annual deaths, GLASS reporting >70% carbapenem-resistant *Klebsiella*/*Acinetobacter*) identifying a need for redistribution of prescriptions to reimburse lower-cost categories and reduce DALYs through optimized defined daily doses (DDDs),^2^,^3^ PMCH shows examples of “low-hanging fruit” such as converting IV to oral medications and using safe and effective alternatives as seen in the 2023 stewardship literature.^10^ There may be gender differences (-140.7 PKR) due to males having more prolonged hospital stays or males receiving more aggressive empiric treatment than females, indicating an opportunity for gender disaggregated AMR data collection under the 2024 Fleming Fund initiative to support AMR surveillance efforts.^16^

Generalizability beyond public rural tertiaries is contingent on many multicenter studies because of the lack of consistent structures between private facilities in Karachi.^10^ Future ASPs must include diagnostic stewardship (which is currently nonexistent at 80% of facilities in Pakistan) to promote the use of culture-directed therapy, producing an estimated 25% more savings overall.^12^,^13^

Overall, the ASP at PMCH establishes a replicable model for pharmacoeconomics in LMICs and should serve as the basis for implementing a coordinated national effort to reduce the cost of antimicrobials through a combination of collaboration between pharmacists and physicians, using a demographic-specific intervention, and implementing metrics-based interventions, with the intention of establishing a policy within the Essential Package of Health Services in Sindh to improve access to antimicrobials.

### Strength of this study:

A salient strength of this study is that it is applicable to a real world. Implementation of the ASP using the existing infrastructure of the hospital and personnel has shown to be feasible and cost effective even in resource limited situations. Consequently, our results make the case for nationwide ASP expansion, especially in rural areas.

### Limitations:

Limitations include a temporal bias caused by seasonal epidemics and the hospital-based perspective which does not take into account the indirect costs of lost productivity, particularly in rural Sindh where 32.8% of the people live in poverty.

## CONCLUSION

This pharmacoeconomic evaluation supports the use of ASPs not only in improving antimicrobial consumption but also reduction in the healthcare expenditure. The cost reduction of 17% for antimicrobial therapy costs attained in this study has significant implications for resource constrained public hospitals.

Demographic variables and, in particular, age and ward type have critical role to cost variation and need to be taken into account in the design for tailoring ASP implementation. The findings have strong support in favor of the inclusion of ASPs in the national health strategies and policies.

### Authors’ Contributions:

**AA:** Designed, Performed the research and article preparation and responsible for the accuracy of the study.

**AA and LA:** Literature search, Study sample and data acquisition.

**AA, MMEAD and LA:** Analyzed data and interpreted data.

**MAG:** Supervised the study, interpreted data, critical review..

All authors have critically reviewed the manuscript and approved the final version.
